# Effects of adjuvant application of corticosteroid and ozone after ultrasound-guided puncture and lavage for the treatment of rotator cuff calcific tendinitis: study protocol for a non-inferiority randomized controlled trial

**DOI:** 10.1186/s13063-023-07401-1

**Published:** 2023-06-05

**Authors:** Jing Dong, Lan Zhang, Haibin Jia, Yuanjiang Zhu, Rui Xiang, Peiyu Li

**Affiliations:** Department of Anesthesiology, Sichuan Provincial Orthopedic Hospital, No. 132 West First Section First Ring Road, Chengdu, China

**Keywords:** Rotator cuff calcific tendinitis, Ozone, Corticosteroid, Randomized controlled trial

## Abstract

**Background:**

Steroid injection after percutaneous irrigation of calcific deposits is a common method for the treatment of rotator cuff calcific tendinitis (RCCT). However, steroids may prevent calcification resorption and cause potentially irreversible damage to tendons. Recent studies have confirmed the positive effects of ozone injection in shoulder tendinopathies, but no RCCTs have been reported. Thus, our study aims to evaluate the non-inferiority of ozone versus steroid injection.

**Methods:**

This is a prospective, randomized, parallel control and non-inferiority trial. A total of 100 patients with unilateral symptomatic RCCT will be enrolled and randomized in a 1:1 ratio to two groups: ultrasound-guided injection with ozone or corticosteroid. The primary outcome is the numeric rating scale for pain (NRS) at 1 week and 3 months following the procedure. Secondary outcomes include a multi-dimensional evaluation of shoulder disability and quality of life improvement, the degree of calcification absorption after treatment, and the number of multiple treatments.

**Discussion:**

The results of this study will provide short-term and long-term evidence for the effectiveness of ozone treatment in RCCT in relieving pain or improving shoulder function.

**Trial registration:**

Chinese Clinical Trial Registry ChiCTR2200063469. Registered on 7 September 2022.

## Administrative information

**Table:** 

Title {1}	Effects of adjuvant application of corticosteroid and ozone after ultrasound-guided puncture and lavage for the treatment of rotator cuff calcific tendinitis: study protocol for a non-inferiority randomized controlled trial
Trial registration {2a and 2b}	ChiCTR2200063469[Chinese Clinical Trial Registry(ChiCTR)] [Registered on 7 Sep 2022] https://www.chictr.org.cn/
Protocol version {3}	Version 3 of 28th March 2022
Funding {4}	This work was supported by the Scientific research project of Chengdu Municipal Health Commission(Project number:2020111)
Author details {5a}	Jing Dong;Sichuan Provincial Orthopedic Hospital, Department of Anesthesiology, Chengdu, China Lan Zhang,Sichuan Provincial Orthopedic Hospital, Department of Anesthesiology, Chengdu, China Haibin Jia,Sichuan Provincial Orthopedic Hospital, Department of Anesthesiology, Chengdu, China Yuanjiang Zhu,Sichuan Provincial Orthopedic Hospital, Department of Anesthesiology, Chengdu, China Rui Xiang,Sichuan Provincial Orthopedic Hospital, Department of Anesthesiology, Chengdu, China Peiyu Li,Sichuan Provincial Orthopedic Hospital, Department of Anesthesiology, Chengdu, China
Name and contact information for the trial sponsor {5b}	Investigator initiated clinical trial;Peiyu Li,(Principal Investigator) lipeiyu2w@163.com
Role of sponsor {5c}	This is an investigator initiated clinical trial. Therefore, the funder played no role in the design of the study; collection, analysis and interpretation of data; and in the decision to write and submit the manuscript for publication

## Introduction

### Background and rationale {6a}

Rotator cuff calcific tendinitis (RCCT) represents an atraumatic shoulder disease characterized by shoulder pain and limited movement, affecting mostly women aged 30 to 60 years [1]. Studies have shown its self-limiting nature, which can be conservatively treated by breaking rest, physical therapy, and oral anti-inflammatory drugs [2, 3].

However, some long-term follow-up studies have shown that tendon calcification is characterized by prolonged pain and reduced activity [4, 5]. Although there is no standard method to treat RCCT, ultrasound-guided puncture and lavage (UGPL) of calcified deposits is widely used in first-line treatment, especially in the acute phase [6].

Subacromial bursa (SAB) steroid injection is a mainstream treatment for managing pain after the procedure [7]. Injection of steroids may reduce inflammation, relieve pain and accelerate functional recovery [8]. Despite these benefits, it has some shortcomings, including the fact that the treatment effect can not be sustained for a long time, the potential for irreversible damage to tendons, and an increased risk of cardiovascular and cerebrovascular accidents [9].

Ozone therapy has been increasingly used for various musculoskeletal disorders in the past four decades because of its bactericidal properties, inflammatory regulation, and circulatory stimulation [10, 11], including low back pain, osteoarthritis, and tendinopathies [12, 13]. Ozone injection has been shown to relieve pain and improve function significantly in shoulder tendinopathies [14, 15], and this positive effect may last for more than 2 months [16]. Furthermore, another study has found that ozone therapy treats acute or chronic tendinitis even in the presence of calcium deposits [17].

Most importantly, there are no reported side effects or damaging adverse reactions to tendons, ligaments, or cartilage using ozone [18]. To our knowledge, there is no randomized clinical study evaluating the impact of ozone injection on RCCT at present.

Based on the above, we hypothesize that the effect of ozone injection is not inferior to that of steroid injection, but the therapeutic effect may last longer. We designed a non-inferiority study to compare the efficacy (pain reduction and disability improvement) of a single SAB injection of ozone with that of a corticosteroid after UGPL for the treatment of RCCT.

### Objectives {7}

The primary aim of this trial is to determine whether SAB ozone injection is as effective as corticosteroid injection in reducing the maximal pain at 1 week (primary time point) and 3 months (secondary time point) following the procedure in patients with RCCT.

Secondary objectives are to compare the shoulder disability and quality of life improvement, the degree of calcification absorption after treatment, the number of multiple treatments, and incidence of adverse events.

### Trial design {8}

The current protocol is a prospective, randomized, controlled, and non-inferiority trial, with two parallel treatment groups (1:1). This study was prospectively registered in China Clinical Trial Registration Center (ChiCTR), with registration number ChiCTR2200063469. The study was conducted in full accordance with the Consolidated Standard of Reporting Trials (CONSORT) [19] and the Standard Protocol Items for Randomized Trials( SPIRIT) [20].

## Methods: participants, interventions, and outcomes

### Study setting {9}

Patients will be recruited in the pain clinic of Sichuan Provincial Orthopedic Hospital. Patients will be considered for inclusion if they meet the criteria outlined below.

### Eligibility criteria {10}

#### Inclusion criteria

- Pain in the unilateral deltoid region
- Worsening symptoms with activities above shoulder level and/or at night
- Positive Hawkins, empty can, and Yocum test results
- Calcifications > 10 mm in size on standard anteroposterior radiographs

### Exclusion criteria

- Age < 18 or > 65 years
- Type 3 calcific deposits according to the classification by Gartner and Heyer [21]
- Comorbidities of the affected shoulder (e.g., glenohumeral arthritis, full-thickness tear of the rotator cuff)
- History of fracture, surgery, previously treated with barbotage or local injection

### Who will take informed consent? {26a}

The attending doctor will screen and record the patients in the outpatient department, and obtain the written informed consent of all eligible patients with a detailed explanation. The study was approved by the Ethics Committee of Sichuan Provincial Orthopedic Hospital(reference KY2022-027–01).

### Additional consent provisions for collection and use of participant data and biological specimens {26b}

On the consent form, participants will be asked if they agree to share their data and whether they agree to continue using their data if they withdraw from the study. This study does not involve collecting biological specimens.

## Interventions

### Explanation for the choice of comparators {6b}

Steroid injection is a mainstream treatment for managing pain after the UGPL.

Injection of steroids may reduce inflammation, relieve pain and accelerate functional recovery. Therefore, we decided to use a compound betamethasone as a comparator.

### Intervention description {11a}

All patients will be irrigated with single-needle technique [22]. Specifically, patients lying supine have a sterile sheet applied after skin antisepsis over the area of interest. Under the guidance of continuous ultrasound, using an in-plane approach, a No. 16 needle will be inserted into the calcification center along the long axis of the tendon after local anesthesia. The needle is connected to a 10 ml syringe with 5 ml of room-temperature normal saline. After gently injecting normal saline into the calcification, the calcific material will reflux back into the syringe with the saline when the piston is released. Repeat this step several times until the backflow saline changes from turbidity to clarity, and pay attention not to damage the calcific margin. The calcification can be slightly fragmented with a syringe needle in the event of difficulty to aspirate at the beginning. Finally, 5 ml of ozone with a concentration of 30 µg/milliliter will be injected into the SAB in the ozone injection (Ol) group [23], while the corticosteroid injection (CI) group received 5 ml mixture solution (the drug composition is compound betamethasone 0.25 ml + vitamin B12 0.5 ml + 2% lidocaine 1 ml + normal saline 3.25 ml) [24]. Finally, an ice pack will be applied to the puncture point for 20 min. All patients will be systematically treated with 0.2 g celecoxib orally for 48 h, avoid strenuous activities, and return for another visit one week later. All interventional procedures will be performed by the same experienced therapist.

### Criteria for discontinuing or modifying allocated interventions {11b}

Participants can withdraw from the study at any time for any reason without consequences. The participant data that have been collected up to that moment will be included in the analysis. Serious adverse events such as infection and tendon laceration at any stage of treatment will be considered for early termination of the study.

### Strategies to improve adherence to interventions {11c}

In this study, specific nurses will be arranged to contact patients regularly to monitor their progress and collect data. If the patient does not return as scheduled, the nurse will contact them by telephone.

### Relevant concomitant care permitted or prohibited during the trial {11d}

New physiotherapy measures are prohibited during the trial, such as acupuncture and extracorporeal shock wave therapy. A rehabilitation program is permitted during the study.

### Provisions for post-trial care {30}

There is no compensation insurance in this study because no serious complications were found in our previous study [24]. The interventions used in this study were considered safe. It is expected that the patient will not suffer personal injury from participating in the trial. If the implementation of the study results in a health hazard to the patient, they will be treated in the appropriate hospital. The cost of treatment will be borne by the patient’s medical insurance.

### Outcomes {12}

#### Primary outcome

The primary outcome is the numeric rating scale for pain (NRS)at 1 week (primary time point) and 3 months (secondary time point) following the procedure [25]. NRS is a commonly used clinical pain assessment tool, which is simple and reliable.

Specifically, take a standard horizontal ruler with a length of 100 mm, and the scale indicates different degrees of pain. On the leftmost side of the level ruler (0 represents “no pain”), the rightmost side (100 represents “the worst pain imaginable”), patients draw marks on the horizontal line according to their painful feelings. The minimum clinically important difference(MCID) in NRS score has been calculated to be 1.6 cm for patients with RCCT [26].

### Secondary outcomes

The secondary outcomes include a multi-dimensional evaluation of shoulder disability and quality of life improvement measured on the Western Ontario Rotator Cuff index (WORC) [27], the University of California at Los Angeles shoulder scale(UCLASS) [28], the Disabilities of the Arm, Shoulder and Hand questionnaire (DASH) [29], the Rotator Cuff Quality-of-life Messure(RC-QOL) [30], the degree of calcification absorption, and the number of multiple treatments and incidence of adverse events.

The WORC is specifically designed to assess shoulder function and quality of life in patients with rotator cuff disorders, consisting of 21 questions divided into five parameters: physical symptoms, sports and recreation, work, social function, and emotional disorders. Each item is in the range of 0 mm (best) to 100 mm (worst). Total score 0 ~ 2100, higher scores indicating worse status.

The UCLASS is a comprehensive evaluation scale with five domains, including pain, function, active forward flexion, forward flexion intensity, and patient satisfaction. Pain and function are scored independently and both range from 1 (worst score) to 10 (best score). The remaining three parameters are given a maximum score of 5 points. Total score range from 2 ~ 35 points. The higher the score, the better the function.

The Dash is a questionnaire to evaluate the impairment degree of affected limbs functional, which contains 30 indicators. Each index has 5 grades from 1 “no difficulty, no symptom, or no impact” to 5 “unable to complete, very severe symptom, or high impact.” The total score is the sum of these 30 indexes, and then convert it to 0–100 points with the following formula:[(sum of score/*n*) − 1] × 25, where *n* is the number of completed responses. A higher score reflects severer disability.

The RC-QOL is a specialized scale to assess the quality of life in patients with rotator cuff disease. The questionnaire includes 34 items covering five areas: symptoms and physical complaints, sports and recreation, work-related concerns, lifestyle issues, and social and emotional issues. Score 0 ~ 100 for each item, summarize the answers of 34 items to form a total score, and then convert to 0 ~ 100 points. A higher score indicates a higher quality of life.

The degree of calcification absorption: using a semi-quantitative evaluation by X-ray as below: no change or minimal changes; the size of calcification decreases by less than 50%; calcification decreases between 50 and 90%; and the size of calcification decreases or disappears by more than 90% [7].

The number of multiple treatments: patients receiving two or more treatments will be recorded. The indication of repeated UGPL procedure is persistent clinical symptoms with calcification deposition, and there is no other cause leading to shoulder discomfort(such as shoulder periarthritis or subacromial bursitis) [31]. A minimum period of each UGPL procedure shall be 4 weeks apart.

The occurrence of adverse events, including abnormal bleeding, infection, and tendon tear, will be recorded throughout the study.

### Participant timeline {13}

**Fig. 1:**
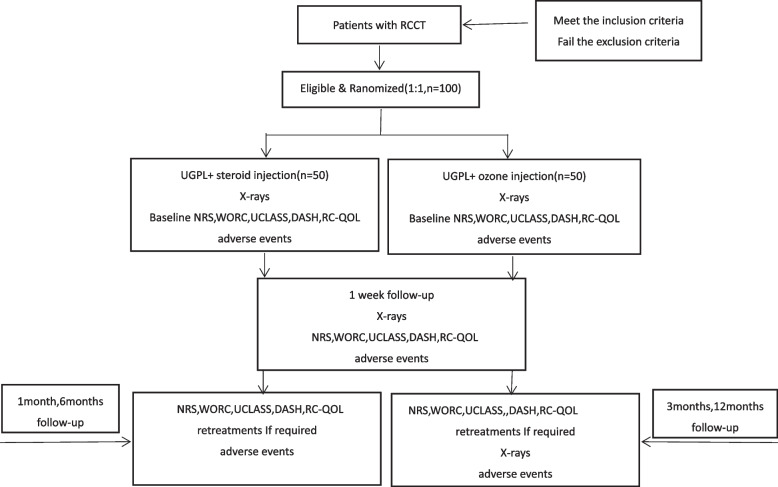
Flowchart of the trial. NRS, numeric rating scale; WORC, Western Ontario Rotator Cuff index; UCLASS, the University of California at Los Angeles shoulder scale; DASH, the Disabilities of the Arm, Shoulder and Hand questionnaire; RC-QOL, Rotator Cuff Quality-of-life Messure

All patients will have five evaluation appointments as follows: pretreatment, at 1 week, 1 month, 3 months, 6 months, and 12 months post-treatment.The detailed schedule for assessments is provided in Table 1, and the flow chart of this study is presented in Fig. 1.

**Table 1:** Schedule of enrolment, interventions, and outcome assessment

	**Study period**						
**Action/timepoint**	Enrolment	**Intervention phase**	**Follow- up**				
			**1 week**	**1 month**	**3 months**	**6 months**	**12 months**
Eligibility screen	×						
Informed consent	×						
History and physical examination	×						
X-rays	×		×		×		×
Randomization	×						
**Interventions**							
UGPL + steroid injection		×					
UGPL + ozone injection		×					
**Assessment**							
NRS	×		×	×	×	×	×
WORC	×		×	×	×	×	×
UCLASS	×		×	×	×	×	×
DASH	×		×	×	×	×	×
RC-QOL	×		×	×	×	×	×
Calcification absorption			×		×		×
Multiple treatments^a^				×	×	×	×
Adverse events		×	×	×	×	×	×

*UGPL* ultrasound-guided puncture and lavage, *NRS* numeric rating scale, *WORC* Western Ontario Rotator Cuff index, *UCLASS* the University of California at Los Angeles shoulder scale, *DASH* Disabilities of the Arm, Shoulder and Hand, *RC-QOL* Rotator Cuff Quality-of-life Messure

^a^If required

### Sample size {14}

We use PASS statistical software to estimate the sample size. The sample size is estimated based on the NRS as the primary outcome measure. The MCID for NRS was defined as 1.6 cm. Earlier research has found that the SD of the NRS is 2.56 points [31]. The one-sided alpha level and statistical power are set to be 2.5% and 80% respectively, each group requires 42 patients. To allow for a 20% rate of loss to follow-up, at least 100 patients will be required to enroll in total.

### Recruitment {15}

The recruitment period runs from 8 September 2022 to 8 April 2023. Patients will be recruited at the pain clinic of Sichuan Provincial Orthopaedic Hospital.

## Assignment of interventions: allocation

### Sequence generation {16a}

Eligible subjects will be randomly divided into the Ol group and CI group in a ratio of 1:1. The random sequence will be obtained by a researcher who does not participate in the treatment and evaluation using the random number function.

### Concealment mechanism {16b}

Each subject will be assigned a unique serial number. Allocation will be hidden in the sequence number within sealed opaque envelopes. Finally, the therapist in the treatment room opens the envelope for the corresponding operation.

### Implementation {16c}

The attending doctor will screen and record the patients in the outpatient department, and obtain the written informed consent of all eligible patients with a detailed explanation. Eligible patients were randomly assigned to one of the study arms, the researchers conducted interventions according to the allocation scheme.

## Assignment of interventions: blinding

### Who will be blinded {17a}

The outcome assessors and data analysts will be blinded to the grouping and intervention given. Due to the different nature and dosage of intervention drugs, the patients and responsible therapist will lack of blinding.

### Procedure for unblinding if needed {17b}

Only the outcome assessors and data analysts will be blinded by the trial design. Therefore, there is no unblinding procedure.

## Patient and public involvement

There was no public or patient involved in the design of this protocol. We encourage the public and patients to actively participate in the whole research process. The feedback of the participants will be collected to improve the study protocol, and they will be informed of the results after the completion of the study.

## Data collection and management

### Plans for assessment and collection of outcomes {18a}

All data of this study will be collected using the case report form, including basic information, baseline evaluation data, and follow-up results. The nurse who is blinded to the grouping and intervention given will complete the follow-up evaluation when patients return for another visit.

### Plans to promote participant retention and complete follow-up {18b}

In this study, specific nurses will be arranged to contact patients regularly to monitor their progress and collect data. If the patient does not return as scheduled, the nurse will contact them by telephone.

### Data management {19}

After all paper case report forms are completed and submitted to the supervisor for review, the data administrator shall input, manage and store the data, which can not be obtained and modified by others. All research data will be archived for 10 years after the study.

### Confidentiality {27}

Study data will be stored using the unique serial number of each participant and locked in the database. All paper case report forms will be locked in the research team’s safe. All research data will be archived for 10 years and then unified destroyed.

### Plans for collection, laboratory evaluation, and storage of biological specimens for genetic or molecular analysis in this trial/future use {33}

There is no plan for the collection, laboratory evaluation, and storage of biological specimens for genetic or molecular analysis in the current trial and for future use in ancillary studies.

## Statistical methods

### Statistical methods for primary and secondary outcomes {20a}

All data of this study will be collected using the case report form, including basic information, baseline evaluation data, and follow-up results. The nurse who is blinded to the grouping and intervention given will complete the follow-up evaluation when the patients return to visit. After all paper case report forms are completed and submitted to the supervisor for review, the data administrator shall input, manage and store the data, which can not be obtained and modified by others. All research data will be archived for 10 years after the study.

We will use SPSS Version 20 (IBM Corp) to analyze the data and the level for statistical difference will be set at 0.05. Chi-square test or Fisher’s exact test will be used to analyze the categorical data. For data conforming to a continuous normal distribution, an unpaired Student’s *t*-test is used to investigate differences between groups, and data not conforming to normal distribution will be analyzed by a nonparametric statistical test.

In order to evaluate whether the OI group is not inferior to the CI group in terms of NRS score decline during the period of follow-up, an intention-to-treat analysis will be conducted. If the lower limit of the one-sided 95% confidence interval of the NRS score of the OI group at the follow-up is within the noninferiority margin (△ = 1.6 points) of the average NRS score of the CI group, it is considered that the OI group is not inferior to the CI group.

A mixed-model analysis with Sidak correction will be performed to analyze the influence of ozone on secondary outcomes. In order to assess whether the statistical differences found are clinically relevant, the differences between groups will be compared with the MCID specific to the questionnaire. Missing data will be disposed of with the multiple imputation method.

### Interim analyses {21b}

There will conduct an interim analysis of the data by the trial leader to confirm the safety and feasibility of the study when half of the test. Serious adverse events such as infection and tendon laceration at any stage of treatment will be considered for early termination of the study.

### Methods for additional analyses (e.g., subgroup analyses) {20b}

There are no subgroup analyses planned.

### Methods in analysis to handle protocol non-adherence and any statistical methods to handle missing data {20c}

The primary and secondary outcomes will be assessed using an intention-to-treat analysis. Missing data will be disposed of with the multiple imputation method.

### Plans to give access to the full protocol, participant-level data and statistical code {31c}

The corresponding authors can be contacted for reasonable access to the full protocol, participant-level dataset, and statistical code.

## Oversight and monitoring

### Composition of the coordinating center and trial steering committee {5d}

The members of the trial steering committee include the principal investigator, the therapist, and the statistician, who are all affiliated with the Department of Anesthesia of Sichuan Provincial Orthopedic Hospital. The principal investigator will be fully responsible for the study and its management. The trial steering committee will be responsible for running the trial day-to-day and providing organizational support. They will meet monthly or more often when necessary to supervise and facilitate the smooth running of the study.

### Composition of the data monitoring committee, its role and reporting structure {21a}

The data monitoring committee is composed of the principal investigator and the nurse. Study nurse will monitor patient data on a weekly basis. Data is delayed or interrupted, the nurse will notify the principal investigator. The data monitoring committee will meet once a year and conduct a rigorous confidential interim review of the trial progress by reviewing and monitoring the trial data, including recruitment quality, data quality, protocol compliance, and adverse events. And it is independent of the sponsor and competing interests.

### Adverse event reporting and harms {22}

Adverse events such as abnormal bleeding, infection, and tendon laceration at any stage of treatment will be recorded. When an adverse event occurs, the investigator should provide an adequate treatment immediately and follow it up until recovery or remission is confirmed.

### Frequency and plans for auditing trial conduct {23}

The research group meets monthly to discuss the progress of the experiment and the problems that will arise. An independent monitor will check the existence and integrity of the investigation documents once a year, including the informed consent, inclusion and exclusion criteria, and source data collection.

### Plans for communicating important protocol amendments to relevant parties (e.g., trial participants, ethical committees) {25}

We do not foresee to amend the protocol. However, if there should be a modification for any reason, we will send a written request for modification permission to the Ethics Committee. Once the amendment is approved, notify the sponsor and funder first, then a copy of the revised protocol will be sent to the principal investigator to add to the Investigator Site File. And update the protocol changes in the clinical trial registry. Finally, any deviations from the protocol will be fully documented using a breach report form.

### Dissemination plans {31a}

The findings will be published in peer-reviewed publications. And the participants will receive a report with all the results of the study.

## Discussion

Up to now, this protocol is the first non-inferiority, randomized, controlled trial comparing the efficacy of a single SAB injection of ozone with that of a corticosteroid after UGPL for the treatment of RCCT. If this study shows that ozone is not only not inferior to steroids in relieving pain or improving shoulder function, but also lasts longer, it can be suggested as an alternative drug for RCCT. UGPL is an effective and low-cost treatment for symptomatic RCCT patients. However, a substantial proportion of patients have severe pain secondary to the inflammatory reaction of calcification aspiration, and numerous patients have recurrent or persistent shoulder symptoms several months following treatment [4, 5]. Some scholars have pointed out that injecting drugs into SAB after UGPL may conduce to prevent acute pain caused by surgery and improve outcomes [7]. Nevertheless,the potential harm of steroids to tendon healing and the negative impact on the calcific deposits’ absorption have also raised some experts’ doubts about their routine use. Currently, many studies have been reported on the treatment of rotator cuff disease with other drugs, including ozone and platelet-rich plasma, but the comparison of different treatment methods is very limited, and even conflicting results have been obtained [32, 33]. Ozone has high oxidation activity, it stimulates angiogenesis and increases circulation and oxygen delivery, while decreasing ischemia, hypoxia, and inflammation at the tissue level, which may contribute to tissue healing and self-renewal [34, 35]. Second, ozone may promote fibroblast and lymphocyte migration to the affected area while decreasing serum levels of certain inflammatory cytokines such as interleukin-1b and tumor necrosis factor- α [36], resulting in the elimination of toxic metabolites produced by inflammation and degeneration. Therefore, it possibly displays more stable antibacterial and anti-inflammatory action compared with steroids. Besides,the anti-inflammatory and analgesic effects of ozone will in turn change the microenvironment and reduction state of hemoglobin, thereby increasing the availability of oxygen and forming a benign cycle [37]. Some studies have noted that ozone can also stimulate endothelial cells and tendon cells to produce a certain biochemical mechanism, inhibit osteoblast differentiation, osteocalcin expression, and calcification of osteoblasts, and accelerates absorption of calcified deposits [38]. Because of these characteristics, ozone therapy has been used in various degenerative and inflammatory musculoskeletal diseases, and it is considered as a complementary and low-risk treatment. Several clinical studies have reported positive results in the treatment of shoulder diseases such as subacromial bursitis, calcified tendinitis, and rotator cuff tears [16, 39]. At present, there is no report of allergy or destructive side effects on tendons or cartilage caused by ozone, which can be safely used in patients with diabetes, hypertension, and so on [9]. What is more, its beneficial effect is sustained for a long time, even for 10 years after intervention [9, 40]. In contrast, despite being effective in the short term, steroid injection for tendon disease is not beneficial in the long term, which may explain the recurrence of shoulder symptoms after UGPL. Thus, through the study, we hope to provide clinicians with high-quality evidence of ozone therapy for RCCT and provide effective treatment methods for such patients.

There are some limitations to this trial. Based on the minimal relevant clinical threshold associated with shoulder pain improvement in previous studies, we select a strict non-inferiority margin of 1.6 points. However, no data are available to define what minimum threshold is clinically meaningful in the case of acute postoperative pain. In this study, the dose and properties (gas or liquid) of the injection drugs in the two groups are different, which is a potentially inevitable limitation.

## Trial status

Recruiting started in September 2022. The current protocol is version 3 of 28 March 2022. It is estimated that patient recruitment will be completed around April 2023.

## Data Availability

The final trial data for this protocol can be supplied on request.

## Abbreviations

RCCT: Rotator cuff calcific tendinitis
NRS: Numeric rating scale
UGPL: Ultrasound-guided puncture and lavage
SAB: Subacromial bursa
ChiCTR: China Clinical Trial Registration Center
CONSORT: Consolidated Standard of Reporting Trials
SPIRIT: Standard Protocol Items for Randomized Trials
Ol: Ozone injection
CI: Corticosteroid injection
MCID: Minimum clinically important difference
WORC: Western Ontario Rotator Cuff index
UCLASS: University of California at Los Angeles shoulder scale
DASH: Disabilities of the Arm, Shoulder and Hand questionnaire
RC-QOL: Rotator Cuff Quality-of-life Messure
